# Genome Sequences of Microviruses Associated with *Coptotermes formosanus*

**DOI:** 10.1128/MRA.00185-19

**Published:** 2019-04-18

**Authors:** Kara Schmidlin, Simona Kraberger, Rafaela S. Fontenele, Francesca De Martini, Thomas Chouvenc, Gillian H. Gile, Arvind Varsani

**Affiliations:** a The Biodesign Center for Fundamental and Applied Microbiomics, Arizona State University, Tempe, Arizona, USA; b School of Life Sciences, Arizona State University, Tempe, Arizona, USA; c Center for Evolution and Medicine, School of Life Sciences, Arizona State University, Tempe, Arizona, USA; d Department of Entomology and Nematology, Fort Lauderdale Research and Education Center, University of Florida, Davie, Florida, USA; e Structural Biology Research Unit, Department of Clinical Laboratory Sciences, University of Cape Town, Observatory, Cape Town, South Africa; University of Delaware

## Abstract

Termites have a unique ability to effectively digest lignocellulose with the help of mutualistic symbionts. While gut bacteria and protozoa have been relatively well characterized in termites, the virome remains largely unexplored. Here, we report two genomes of microviruses (termite-associated microvirus-1 [TaMV-1] and termite-associated microvirus-2 [TaMV-2]) associated with the gut of Coptotermes formosanus.

## Abstract (precis)

Termites have a unique ability to effectively digest lignocellulose with the help of mutualistic symbionts. While gut bacteria and protozoa have been relatively well characterized in termites, the virome remains largely unexplored.

## ANNOUNCEMENT

**FIG 1:**
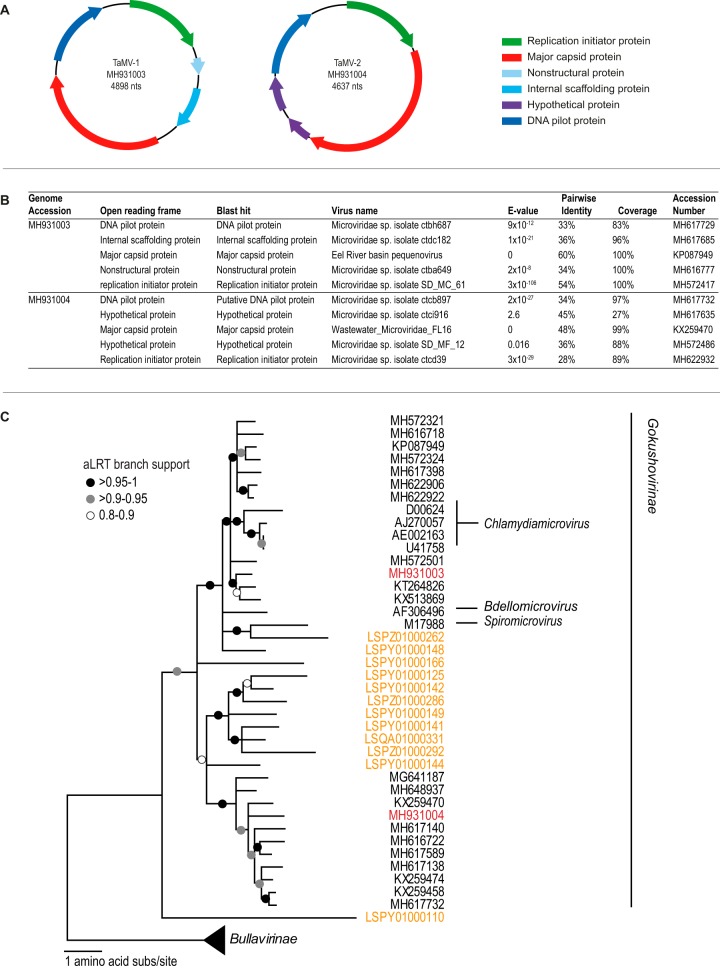
(A) Genome organization of termite-associated microvirus-1 (replication initiator protein, 882 nucleotides [nt]; nonstructural protein, 276 nt; internal scaffolding protein, 468 nt; major capsid protein, 1,704 nt; and DNA pilot protein, 837 nt) and termite-associated microvirus-2 (replication initiator protein, 1,017 nt; major capsid protein, 1,608 nt; hypothetical proteins, 339 and 417 nt; and DNA pilot protein, 768 nt). (B) Summary of the best BLASTp results for each ORF of TaMV-1 and TaMV-2. (C) Maximum likelihood phylogenetic tree of the MCP amino acid sequences and the pairwise identities of the MCP of most closely related *Gokushovirinae* members, those from termite reported by Tikhe and Husseneder (5), and those from this study. Numbers in red are MCP sequences from this study, and numbers in orange are MCP sequences identified in termites by Tikhe and Husseneder (5). The maximum likelihood phylogenetic trees were inferred with PhyML (17) with the RtRev+F+G substitution model and with approximate likelihood ration test (aLRT) branch support.

The Formosan subterranean termite Coptotermes formosanus is native to China but is invasive in various subtropical areas around the world. It is an economically important species that forms large colonies and causes extensive damage to a variety of wood types (1, 2). In order to break down lignocellulose of woody plants and acquire essential nutrients, termites rely on a diverse range of hindgut symbionts, including bacteria and protozoa (3, 4). While the relationship between termites and their symbiotic gut community has been examined, the viral community remains largely unknown. Recently, 13 novel bacteriophages associated with C. formosanus and four novel genomoviruses with fungus-farming termites (Odontotermes spp.) were identified (5–7). To further characterize termite viruses, 10 *C. formosanus* gut samples were collected, pooled, and homogenized in 200 µl SM buffer (100 mM NaCl, 8 mM Mg_2_SO_4_, 0.01% gelatin, 50 mM Tris-HCl; Teknova, USA). The homogenate was used for viral DNA extraction, as previously described (8–10). Circular molecules were enriched by rolling circle amplification using TempliPhi 100 amplification (GE Healthcare, USA), and the resulting DNA was used to construct a 2 × 150-bp library using the Illumina TruSeq Nano DNA library prep kit and sequenced on an Illumina HiSeq 4000 platform at Macrogen, Inc. (South Korea). The raw paired-end reads (36,773,486 in total) were trimmed using Trimmomatic (11) and then *de novo* assembled using metaSPAdes 3.11.1 (12), with k-mer values of 33, 55, and 77. In the resulting 102,367 contigs (*N*_50_, 1,491 nucleotides [nt]), a 4,975-nt contig (with 176× coverage) and a 4,714-nt contig (with 66× coverage) were identified as having similarities to microvirus sequences using BLASTx (13). Microviruses are prokaryote-infecting viruses with small circular single-stranded DNA genomes (14) that are packaged in icosahedral capsids (15). Within the family *Microviridae*, there are two subfamilies, *Bullavirinae,* whose members infect mainly *Enterobacteria,* and *Gokushovirinae,* whose members infect obligate intracellular parasitic bacteria (16). The genomes of termite-associated microvirus-1 (TaMV-1; GenBank accession number MH931003) and termite-associated microvirus-2 (TaMV-2; GenBank accession number MH931004) have genome organizations similar to those of other gokushoviruses (Fig. 1A and B), and phylogenetic analysis of the major capsid protein (MCP) confirms that both microviruses group with other members of this subfamily (Fig. 1C). TaMV-1 MCP shares ∼60% amino acid identity with the MCP of the microvirus with accession number KP087949, whereas the TaMV-2 MCP shares ∼48% amino acid identity with the MCP of the microvirus with accession number KX259470 (Fig. 1B). A data set of the MCPs of all published microviruses was assembled and used to query the top 10 BLASTp hits to the MCPs of TaMV-1 and TaMV-2 (Fig. 1B). These 20 MCPs, together with those from this study, those from termites reported by Tikhe and Husseneder (5), and those of classified microviruses were used to infer a maximum likelihood phylogenetic tree using PhyML (17). The MCP amino acid sequences of TaMV-1 and TaMV-2 share 36% pairwise identity with each other (Fig. 1C), with TaMV-1 clustering with MCPs of microviruses in the genus Chlamydiamicrovirus, whereas TaMV-2 clusters with those of unclassified microviruses. TaMV-1 and TaMV-2 are distinct from the microviruses identified by Tikhe and Husseneder (5), sharing <41% MCP amino acid identity. This highlights that there are diverse microviruses inhabiting the termite gut, and future work is needed to determine the role these viruses play in the complex host-symbiont interaction.

### Data availability.

The complete genome sequences of termite-associated microvirus-1 (TaMV-1) and termite-associated microvirus-2 (TaMV-2) isolates are deposited in GenBank with accession numbers MH931003 and MH931004, respectively. Raw reads have been deposited in the Sequence Read Archive (SRA) with accession number PRJNA521362.
